# 

**Published:** 1893-07

**Authors:** 


### BOOKS RECEIVED.

Napheys' Modern Therapeutics, Medical and Surgical, including the Diseases of Women and Children. A compendium of recent formulæ and therapeutical directions from the practice of eminent contemporary physicians, American and foreign. Ninth edition, revised and enlarged. Volume II. General Surgery, Gynecology, and Obstetrics. By Allen J. Smith, M. D., Professor of Pathology, University of Texas, Galveston; late Assistant Demonstrator of Morbid Anatomy and Pathological Histology, and Lecturer on Urinology, University of Pennsylvania, and J. Aubrey Davis. M. D., Assistant Demonstrator of Obstetrics, University of Pennsylvania; Assistant Physician to Home for Crippled Children, Philadelphia. Large octavo volume. Pp. 19-1112. Price, $6. Philadelphia: P. Blakiston, Son & Co., 1012 Walnut street. 1893.

A Hand-book of Local Therapeutics. General Surgery. By Richard H. Harte, M. D., Demonstrator of Osteology and Syndesmology, University of Pennsylvania; Surgeon to the Episcopal and St. Mary's Hospitals; Consulting Surgeon to St. Timothy's Hospital. Diseases of the Skin. By Arthur Van Horlingen, M. D., Professor of Diseases of the Skin in the Philadelphia Polyclinic and College for Graduates in Medicine; late Clinical Lecturer on Dermatology in Jefferson Medical College; Dermatologist to the Howard Hospital. Diseases of the Ear and Air-passages. By Harrison Allen, M. D., Consulting Physician to the Rush Hospital for consumption; late Surgeon to the Philadelphia and St. Joseph's Hospitals. Diseases of the Eye. By George C. Harlan, M. D., Surgeon to Wills' Eye Hospital and to the Eye and Ear Department of the Pennsylvania Hospital; Emeritus Professor of Diseases of the Eye, Philadelphia Polyclinic, etc. Edited by Harrison Allen, M. D. Octavo, pp. xxvii.—505. Price, $4. Philadelphia: P. Blakiston, Son & Co., 1012 Walnut street. 1893.

The Surgical Anatomy and Surgery of the Ear. By Albert H. Tuttle, M. D., S. B., (Harv.) of Cambridge, Mass.; Member of the Massachusetts Society; Member of the American Medical Association; Secretary of the Boston Gynecological Society; Secretary of the Cambridge Medical Improvement Society; Member of the Harvard Medical School Association; Member of the Lawrence Scientific School Association, etc. With twenty-eight original illustrations, reproduced from the writer's drawings from Nature. Physicians' Leisure Library, issued monthly; single copies, 25 cents. Detroit: George S. Davis. 1892.

Technique d'Électrothérapie. G. Gautier, J. Larat, Maloine, Editeur, 91 Boulevard Saint-Germain, Paris.

International Clinics. A Quarterly of Clinical Lectures on Medicine, Neurology, Pediatrics, Surgery, Genito-Urinary Surgery, Gynecology, Ophthalmology, Laryngology, Otology, and Dermatology. By professors and lecturers in the leading medical colleges of the United States, Great Britain, and Canada. Edited by John M. Keating, M. D., LL. D., Colorado Springs, Col.; Fellow of College of Physicians, Philadelphia; formerly Consulting Physician for Diseases of Women to St. Agnes' Hospital; Gynecologist to St. Joseph's Hospital; Visiting Obstetrician to the Philadelphia Hospital, and Lecturer on Diseases of Women and Children, Philadelphia; Editor Cyclopedia of the Diseases of Children. Judson Daland, M. D., Philadelphia; Instructor in Clinical Medicine, and Lecturer on Physical Diagnosis and Symptomatology in the University of Pennsylvania; Assistant Visiting Physician to the University Hospital; one of the Examiners of the Insane to the Philadelphia Hospital; Visiting Physician to St. Clement's Hospital, Philadelphia. J. Mitchell Bruce, M. D., F. R. C. P., London, England, Physician and Lecturer on Therapeutics at the Charing Cross Hospital. David W. Finlay, M. D., F. R. C. P., Aberdeen, Scotland, Professor of Practice of Medicine in the University of Aberdeen; Physician to, and Lecturer on Clinical Medicine in the Aberdeen Royal Infirmary; Consulting Physician to the Royal Hospital for Diseases of the Chest, London. Volume I. Third series. Royal octavo, pp. xi.—356. Philadelphia: J. B. Lippincott Co. 1893.

Lessons in Physical Diagnosis. By Alfred M. Loomis, M. D., LL. D., Professor of the Practice of Medicine and Pathology in the University of the City of New York. Tenth edition, revised and enlarged. Octavo; illustrations, some in color; 240 pages, extra muslin; price, $3. New York: William Wood & Co.

Appendicitis and Perityphlitis. By Carlton Talamon, M. D., Physician to Tenon Hospital, Paris, France. Translated by E. P. Hurd, M. D. Physicians' Leisure Library. Single copies, 25 cents; subscription price, $2.50 a year. Detroit: George S. Davis. 1893.

A Practical Treatise on Materia Medica and Therapeutics, with Especial Reference to the Clinical Application of Drugs. By John V. Shoemaker, A. M., M. D., Professor of Materia Medica, Pharmacology, Therapeutics, and Clinical Medicine, and Clinical Professor of Diseases of the Skin in the Medico-Chirurgical College of Philadelphia; Physician to the Medico-Chirurgical Hospital; Member of the American Medical Association, of the Pennsylvania and Minnesota State Medical Societies, the American Academy of Medicine, the British Medical Association; Fellow of the Medical Society of London, etc., etc. Second edition, revised. In two royal octavo volumes. Volume I., 353 pages, devoted to Pharmacy, General Pharmacology, and Therapeutics and Remedial Agents not Properly Classed with Drugs. Volume II., 680 pages. An independent volume upon Drugs. Volume I., in cloth, $2.50 net; sheep, $3.25 net. Volume II., in cloth, $3.50 net; sheep, $4.50 net. Philadelphia: The F. A. Davis Company, publishers. 1914 and 1916 Cherry street.

The Year-Book of Treatment for 1893. A Critical Review for Practitioners of Medicine and Surgery. A series of contributions by twentytwo writers. In one 12mo volume of 500 pages. Cloth, $1.50. Philadelphia: Lea Brothers & Co. 1893.

Transactions of the American Gynecological Society. Volume XVII. For the year 1892. Small octavo, pp. xxxix.—493. Philadelphia: William J. Dornan, printer. 1892.

Transactions of the Kentucky State Medical Society. New series. Volume I. Thirty-seventh annual meeting, held at Louisville, May 4, 5, and 6, 1892. Octavo volume, pp. x.—340. Louisville: Printed by John P. Morton & Company. 1892.

A Text-book of Practical Therapeutics, with Especial Reference to the Application of Remedial Measures to Disease and their Employment upon a Rational Basis. By Hobart Amory Hare, M. D., B. Sc., Professor of Therapeutics and Materia Medica, in the Jefferson Medical College, of Philadelphia; Physician to St. Agnes' Hospital, and to the Jefferson Medical College Hospital; Consulting Physician to the Franklin Reformatory Home; Laureate of the Royal Academy of Medicine, in Belgium; of the Medical Society of London, etc. President of the Section of Therapeutics in the Pan-American Medical Congress. Third edition, enlarged and thoroughly revised. Octavo volume, pp. xiii--696. Philadelphia: Lea Brothers & Co. 1892.

Hand-book of Massage, by Emil Kleen, M. D., Ph. D., Practicing Physician in Carlsbad, Bohemia. Authorized translation from the Swedish. By Edward Mussey Hartwell, M. D., Ph. D., Director of Physical Training in the Public Schools of Boston; Late Associate in Johns Hopkins University. Octavo volume, pp. xiv.—316. Price, $2.75. Philadelphia: P. Blakiston, Son & Co., 1012 Walnut street. 1892.

Recherches Cliniques and Therapeutiques sur L'Épilepsie, L'Hystérie et L'Idiotie. Compte rendu du service des enfants idiots, épileptiques, et arriérés de bicetre pendent l'année 1891. Par Bourneville, medecin de bicetre. Avec la collaboration de MM. Banzet, Finet, Ishwall, Raoult, A. Sorel et P. Sollier. Internes et anciens internes du service. Volume XII., avec fourteen figures dans le texte et two plaches. Paris: aue Bureau du Progres Medical, 14 rue des Carmes. Vve. Babé et Cie, editeurs, Place de l'École de Medecine. 1892.

A Text-book of the Theory and Practice of Medicine. By American teachers. Edited by William Pepper, M. D., LL.D., Provost and Professor of the Theory and Practice of Medicine and of Clinical Medicine in the University of Pennsylvania. In two volumes; illustrated. Vol. I. Large octavo, pp. 909. Philadelphia: W. B. Saunders, 913 Walnut street. 1893.

System of Diseases of the Ear, Nose, and Throat. Edited by Charles H. Burnett, A. M., M. D., Emeritus Professor of Otology in the Philadelphia Polyclinic; Clinical Professor of Otology in the Woman's Medical College of Pennsylvania; Aural Surgeon to the Presbyterian Hospital, etc., Philadelphia. Vol. I. Illustrated. Philadelphia: J. B. Lippincott Co. 1893.

---

NOTICE TO CONTRIBUTORS.—We are glad to receive contributions from every one who knows anything of interest to the profession. Articles designed for publication in the JOURNAL should be handed in before the first day of the month. The Editors are not responsible for the views or opinions of contributors. All communications should be addressed to the Managing Editor, 284 FRANKLIN ST., BUFFALO, N. Y.

### INDEX.

PAGE.

ABSTRACTS . . 489, 559, 625, 676
Abscess, Intracranial, from Aural Disease: Operations for . . . . . . . . . . . . . . 623
Hepatic with Gallstones . . 612
Academy of Medicine Notes . . . . . . . . . . . . . . . 570, 632, 696
Actinomycosis, With Report of a Case . . . . . . . . . . 325
Adenoma of the Naso-pharynx in Relation to Bronchitis and Asthma . . . . . . . . . . . . . . . 581
Advertising by Physicians . . . . 559
Antiseptics, Poisoning by . . . . 559
Angio-neurotic Edema . . . . . . 488
Address of the President, Annual, before the Medical Society of the County of Erie . . 401
Decennial, Niagara University Commencement . . . 645
Appendicitis, Treatment of, With Illustrative Cases . . . . . . . 1
Arsenic in Chorea . . . . . . . . 111
Asphyxia, Intrauterine . . . . . . 640
Asthma, Treatment and Management of . . . . . . . . . . . . 288
Asylum Physicians, Are they Party Pensioners ? . . . . . . . . . . 679
Atelectasis, Congenital . . . . . . 610

BACTERIOLOGY and Dermatology, Department of . . . . 167
Bacteriologist to the Health Department . . . . . . . . . . . . . 562
Bacillus of the Rhinoscleroma . . 170
Banknote . . . . . . 580
Banknotes, Disinfection of . . . . 580
Bartlett, Frederick W., M. D., Intestinal Irrigation—Enteroclysis. 542
Benedict, A. L., M. D., Sixth Health District of Buffalo, Condition of . . . . . . . . . . . . 173
Best Code . . . . . . . . . . . . 241
Bichloride and Germs . . . . . . 541
Blood in Melancholia and Effect of Systematic Tonic Treatment . . 618
Blood Poisoning and Cases of Assault . . . . . . . . . . . . 301
Books and Pamphlets Received . . 125, 195, 259, 323, 386, 451, 511, 576, . . . . . . . . . . . . 641, 708, 770

PAGE.

Bovine Consumption and the Milk Supply . . . . . . . . . . . 626
Brain Injuries, Symptoms of . . . 559
Browning, William, M. D., Nervous Affections that may arise from Malaria . . . . . . . . . 676
Bread and Dyspepsia . . . . . . . 491
Bruises of the Brain . . . . . . . 104
Buffalo Health Department . . . . 173
International Expositon . . 55
Medical and Surgical Journal . . . . . . . . . . . . 541

CARBUNCLE, Hydrogen Peroxide in the Treatment of . . 673
Carica Papaya in Gastro-intestinal Disorders . . . . . . . . . . . 97
Carmer, Lewis C., M. D., Memoir of . . . . . . . . . . . . . . 430
Cary, Charles, M. D., History and Present Standing of the University of Buffalo . . . . . . . . . 519
Care of the Person in Relation to Cholera . . . . . . . . . . . . 178
Cerebellum, a Cyst of the . . . . 748
Cerebral Implication in Otorrhea, Symptoms of . . . . . 622
Surgery, Contributions to . 490
Children's Rights . . . . . . . . 709
Chlorosis . . . . . . . . . . . . 50
Chloroform in Typhoid Fever . . . 50
Chorea, Treatment of by Exalgine . 109
by Arsenic . 111
Cholera . . . . . . . . . . . . . 171
Care of the Person in Relation to . . . . . . . . 178
Precautions Against . . . . 176
and the Law . . . . . . . 180
Civil Service Examinations . . 68, 437
Clark, Horace, M. D., Adenoma of the Naso-pharynx in Relation to Bronchitis and Asthma . . . . . 581
Clinical Reports . . . . . . . . . 547
Code, Best . . . . . . . . . . . 241
College Commencements . . . . . 684
Columbian Exposition . . . . . . 692
Colorado and Phthisical Patients . 20
Constipation and other Disorders of the Intestines: Treatment by Olive Oil Enemata . . . . . . . . 666
Congenital Atelectasis . . . . . . 610

PAGE.

**Correspondence:**

Letter to Ferd. C. Valentine from Dr. Roswell Park . . . 431
Letter to Dr. Roswell Park from Ferd. C. Valentine . . 432
Letter from Prof. Czerny to Claudius H. Mastin, M. D. . 492
Letter from Chas. A. L. Reed, M. D., to Prof. Czerny . . . 494

Crockett, M. A., M. D., Gonococcus In Its Relation to Ascending Gonorrhea in Women . . . . . . . 465
Cushing, Clinton, M. D., Endometritis . . . . . . . . . . . . . . 283
Cyst of the Cerebellum . . . . . . 748

DAGENAIS, Alphonse, M. D., A Case of Congenital Atelectasis . . . . . . . . . . . . 610
Daggett, B. H., M. D., Irrigation of the Urethra and Bladder by Posture and Continuous Current . 453
Daily Exercise, Some Reasons for . 682
Decennial Address, Annual Commencement Niagara University 645
Department of Health, Annual Banquet . . . . . . . . . . . . . 499
Diphtheria, Papoid in . . . . . . 11
Dowd, J. Henry, M. D., Chronic Gonorrhea, Causes and Treatment of . . . . . . . . . . . . 660
Duboisine in Paralysis Agitans . . 681
Dunham, Sydney A., M. D., Gallstone with Hepatic Abscess . . . 612

ECTOPIC GESTATION, Actual Experience with Cases . . . 21

**Editorials:**

American Pharmaceutical Association . . . . . . . . 371
Text-Book of Surgery . . 501
Buffalo Department of Health . 370
Bureau of Hygiene and Sanitation . . . . . . . . . . 308
Carnegie Laboratory . . . . . 500
Church Bells . . . . . . . . 499
Eastern Michigan Asylum for the Insane, Report of Trustees . . . . . . . . . . . . . 435
Government of Venezuela and the Pan-American Medical Congress . . . . . . . . . 501
International Medical Annual for 1893 . . . . . . . . . . 501

PAGE.

Medical Society of the County of Erie . . . . . . . . 434
Society of the State of New York . . . . . . 497
Michigan State Board of Health 114
Mississippi Valley Medical Association . . . . . . . . . . 242
New York State Reformatory, Sixteenth Year Book of . . . 115
Pan-American Medical Congress . . . . . 246, 305, 567, 568
Paper Money for Transmission of Bacteria . . . . . . . . 433
Prizes, the Hodgkins Fund . . 758
Pullman Sleeper . . . . . . . . 433
Railway Surgery of the Pan-American Medical Congress, Section of . . . . . . . . . . 308
Report on Insanity, Clinical . 500
Report of the Chief of Bureau of Medicine and Surgery . . 436
Roads, Question of Good . . . 372
Sheppard Asylum and Hospital . . . . . . . . . . . . . 757
State Commission in Lunacy . 373
Society Meetings and Transactions for 1892 . . . . . . . . 369
Southern Pines Resort Co. . . 372
Surgical and Gynecological Association . . . . . 307
Surgery and Surgeons of Virginia, Prize for History of . 183
The Relation of Noise to Good Health . . . . . . . . 754
Times and Register, Letter to by Joseph Price, M. D. . . . 114
United States Pharmacopeia . 309
Warming Street Cars in Winter . . . . . . . . . . . . . 437
Electricity in the Diagnosis of Diseases of the Nervous System . . . . . . . . . 148
In the Treatment of Diarrhea and Cholera . . . 486
Endometritis, Clinical Lecture on . 386
Treatment of . . . . . . 599
Epilepsy, Treatment of . . . . . . 12
Evans, Z. H., M. D., The Nature of and the Choice of Methods in the Treatment of Uterine Cancer . . . . . . . . . . . . . . 653
Eyelids, Vaccinia of . . . . . . . 364
Eye, Legislation for Protection of . 301
Paralysis . . . . . . . . . . . 620

FEMALE PHYSICIANS in State Hospital Service, Open Competitive Examination for Candidates . . . . . . . . . . . 578

PAGE.
Fractures, Vertebral . . . . . . . 625
Frederick, C. C., M. D., Treatment of Gonorrhea and Gonorrheal Disease in Women . . . . . . . . 470
Nervous Disturbances of Pregnancy Due to Defective Excretion . . . . . 603
Fresh Air Mission . . . . . . . . 691

GALL-STONE with Hepatic Abscess . . . . . . . . . . 612
Gall-bladder Surgery, Present Position of . . . . . . . . . . . . 628
Gastro-intestinal Disorders, Carica Papaya . . . . . . . . . . . . 97
Gastric Disorders, Diagnosis of . . 535
Genital Irritation, Relation of, to Nervous and Mental Diseases . . 485
Germany and the Pan-American Medical Congress . . . . . . . 492
Gestation, Ectopic, Experience in . 21
Gibney, V. P., M. D., Suppuration Complicating Tuberculous Disease of the Bones and Joints, Management of . . . . . . . . 680
Gonococcus, Its Relation to Gonorrhea in Women . . . . . . . . 465
Gonorrhea and Gonorrheal Pelvic Disease in Women, Treatment of . . . . . 470
Its Relation to Pelvic Diseases in Women . . . . 459
Chronic, Causes and Treatment of . . . . . . . . 660
Grimaux, E., Quinine, Double Salts of, etc. . . . . . . . . . . 362
Gude's Manganiferous Iron Peptone, Uses and Effects of . . . . 295
Gyroma and Endothelioma of the Ovary, Diagnosis and Clinical Aspects of . . . . . . . . . . . 197

HAIR, Medicine for the . . . . 131
Hamburg-American Packet Steamship Co . . . . . 181
Hayd, Herman E., M. D., Endometritis, Treatment of . . . . . . 599
Heart Failure . . . . . . . . . . . 627
Motor Neurosis of . . . . . 619
Heitzmann, M. D., Gude's Manganiferous Iron Peptone . . . . . 295
Himmelsbach, G. A., M. D., Syphilis and Marriage, Translation . . . . . . . . . . 417
Constipation and Other Disorders of the Large Intestine: Treatment by Olive Oil Enemata, Translation . . . . . . 666

PAGE.
History and Present Standing of the University of Buffalo . . . . 519
Homes, Professional . . . . . . . 367

**Hospital Notes:**

Buffalo City Hospital—Positions of House Surgeon and Assistant Vacant . . 375
General Hospital . . . . . 696
Hospital Sisters of Charity 696
Emergency Hospital . . . . . 696
Fitch Hospital . . . . . . . . 696
Riverside Hospital for Women 563
St. Clement's Hospital . . . . 502

Hot Baths, Influence of on the Excretion of Nitrogen and Uric Acid from the Human Body . . . 555
Hubbell, Alvin A., M. D., Extraction of Steel from the Interior of the Eye . . . . 389
Ophthalmology . . . . . 364
Treatment of Marginal Blepharitis . . . . . . 621
Hydrochloric Acid in Vomiting . . 479
Hydrogen Peroxide in the Treatment of Carbuncle . . . . . . . 367
Hypnotics . . . . . . . . . . . . 618
Hypodermic Sepsis, Danger of . . 51
Hysteria with Gastric Ulcer . . . 166

ILLINOIS State Board of Health . . . . . . . . . . . . . 112
Immigration Laws, Defects of . . 302
Impotence, Hypnotic Suggestion for . . . . . . . . . . . . . . . 110
Incompatibilities in Prescriptions . 298
Incontinence of Urine, and Phimosis—Nocturnal . . . . . . . . . 487
Indigestion and Malnutrition in Nursing Infants . . . . . . . . 726
Infants, Causes of Indigestion and Malnutrition in Nursing . . . . 726
Infection, Puerperal . . . . . . . 17
Insomnia, Treatment of . . . . . 110
Instruments, to make Steel, as bright as new . . . . . . . . . 580
Internal Medicine . . . . . . . . 50
Intrapelvic Diseases of Women Early Diagnosis, and Early Operation in . . . . . . . . . . . 76
Intestinal Irrigation—Enteroclysis. 542
Intra-cranial Affection, New Sign in Diagnosis of . . . . . . . . 484
Intrauterine Asphyxia . . . . . . 740
Ischio-rectal Abscess and Fistula-in-ano, Treatment of . . . . . . 352

PAGE.

Itching of Central Origin, or Brain Itching . . . . . . . . . . . 617
Iodide of Strontium in Cardiac and other Affections . . 480
Potassium in Pott's Paraplegia . . . . . . . . . 665

JONES, ALLEN A., M. D., Hysteria with Gastric Ulcer . . . . . . . . . . 166
Diagnosis of Gastric Disorders . . . . . . . . . . 535
Jones, Mary A. Dixon, M. D., Ovarian Tumor . . . . . 292
Gyroma and Endothelioma of the Ovary, Diagnosis and Clinical Aspects of . 197

**Journalistic Notes:**

Annals of Ophthalmology and Otology . . . . . . . . . . 246
Bacteriological World and Modern Medicine . . . . . 563
Brooklyn Medical Journal . . 183
Buffalo Commercial . . . . . 439
Charlotte Medical Journal . . 183
Chicago Medical Record . . . 132
Electrical Review . . . . . 244
Hot Springs Medical Monthly . 185
Indiana Medical Journal . . . 246
International Medical Annual for 1893 . . . . . . . . . . 438
Medical Review . . . . . . . 310
Medical and Surgical Observer 367
Medical Compend . . . . . . 438
Medical Bulletin . . . . . . . 439
New York Medical Times . . . 115
New York Polyclinic . . . . . 563
Ontario Medical Journal . . . 117
Revista Médico-Quirurgicana Americana . . . . . . . . 245
Sanitarian . . . . . . . . . . . 246
Weekly Bulletin of Newspaper and Periodical Literature . . 118

**Journalistic and Literary Notes:**

Alumni Association, Harvard Medical School, Fourth Bulletin . . . . . . . . . . . . 759

Judson, A. B., M. D., Pathology and Treatment of Club-foot . . . 155

KIDNEY, Operations on . 338
Koch, Laboratory at World's Fair . . . . . . . . . . . 605

Krauss, W. C., M. D., Chorea, Treatment of by Exalgine . . . . . . . . . . . 109
Tendon Reflexes, Behavior of, in Health and Disease . . . . . . . . . . 133
Lithemia, Discussion of . 215
Trional as an Hypnotic, Value of . . . . . . . . 483
Neuritis of both Sciatic Nerves, Report of a Case of . . . . . . . 552
Kurtz, Joseph, M. D., Vertebral Fractures . . . . . . . . . . . 625

LABOR, Neuritis and Myelitis and the Forms of Paralysis and Pseudo-paralysis following . . . . . . . . . . 750
Laborde and Malbec, M. D., Iodide of Strontium in Cardiac Affections . . . . . . . . . 480
Laceration of Cervix, Endometritis, Lateral Displacement of Uterus, Pelvic Peritonitis following Abortion . . . . . . . . . . . . . 549
Larynx, Ulceration of, following Intubation . . . . . . . . . . . 51
Legislation for Protection of the Eye . . . . . . . . . . 301
Introduction of Southworth Bill . . . . . . . 629
Leucomaine, a new . . . . . 555
Lichen Rubor Planus, complicated by Pemphigus Exfolacius . . . . 168
Life Underwriters' Association of Western New York . . . . . . . 691
Lithemia, Discussion of . . . . . 215
Digestive Disturbances in . 229
Clinical Aspects of . . 220
Literary: Harvard Medical School Association, Second Bulletin . . . . . . . . . 56
Third Bulletin . . . . . .
Lothrop, Thomas, M. D., Decennial Address, Niagara University 645
Love, I. N., M. D, Valedictory Address, Marion-Sims College of Medicine . . . . . . . . . . . . 709

McMURTRY, L. S., M. D., Minor Gynecological Operations, Relations of to Intrapelvic Inflammation . . 69
Malaria, Nervous Affections that May Arise from . . . . . . . . 676
Manley, Thomas H., M. D., Osteogenesis and Osteoplasty in Crushing Lesions of the Extremities . . 261

PAGE.

Mann, Matthew D., M. D., Relation of Gonorrhea to Pelvic Disease in Women . 459
Laceration of Cervix, Endometritis, Lateral Displacement of Uterus, Pelvic Peritonitis following Abortion . . . . 549
Marcy, William L., Esq., Responsibility of Physician to Patient, Considered from a Legal Point of View . . . . . . . . . . . . . 606
Mays, Thomas J., M. D., Asthma, Treatment and Management of 288
Maxillary Nerve, Removal of . . . 293
McAdam, L. J., M. D., A Cyst of the Cerebellum . . . . . . . 748
Medical Jurisprudence, Recent . . 299
Education in the Southern States . . . . . . . . . 240
Spelling and Pronunciation . . . . . . . . 145
And Surgical Register of the United States . . 132
Medicine, Specialism in, as Related to Surgery and Gynecology . . . . . . . . . . 302
For the Hair . . . . . . 131

**Medical College Notes:**

Buffalo University . . . . . . 184
University . . . . . . . . 517
University, Examinations . 571
University . . . . . . . . 634
University of Buffalo . . . . . 562
Of Buffalo . . . . . . . . 687
Niagara University . . . . . . 184
University . . . . . . . . 571
University . . . . . . . . 635
University . . . . . . . . 684

Memoir of Lewis C. Carmer, M.D. 430
Mercer, A. Clifford, M. D., Congenital Syphilis and Rickets, Clinical Remarks on . . . . . 459
Method of Regulating the Practice of Medicine . . . . . . . . . . . 113
Meyers, R. S., M. D., Memoir of Lewis C. Carmer, M. D. . . . . 430
Milliken, Samuel E., M. D., Iodide of Potassium in Pott's Paraphlegia . . . . . . . . . . . . . . 665
Miller, John A., M. D., Excretion of Nitrogen in Urine . . . . . . 554
Mills, Charles K., M. D., Neuritis and Myelitis and the Forms of Paralysis and Pseudo-paralysis following Labor . . . . . . . . 750

PAGE.

Minor Gynecological Operations, Relation of to Intrapelvic Inflammation . . . . . . . . . . . 69
Morphine, Are Hypodermic Injections of Lawful? . . . . . . . . 300
Muscular Atrophy, Progressive . . 616

NATIONAL ASSOCIATION of Railway Surgeons . . . . 116
National Quarantine . . . . . . . 192
Law, New . . . . . . . . . 560
Neuritis, Pressure, of both Sciatic Nerves, Report of a Case . . . . . . . . . . 552
and Myelitis and the Forms of Paralysis and Pseudo-paralysis following Labor . . . . . . . . . . 750
Neurosis of the Heart, Motor . . . 619
New York State Law Controlling the Practice of Medicine . . . . . . . . . . . 128
Reformatory at Elmira . . 692

**New Instruments:**

Shield to Protect the Clinical Thermometer . . . . . . . 683

**Obituary:**

Coles, Walter, M. D. . . . . . . 248
Fisher, George Jackson, M. D. 502
Harding, E. G., M. D. . . . . . 60
Jackson, A. Reeves, M. D. . . 374
Johnson, Laurence, M. D. . . 564
Lee, Charles Carroll, M. D. . 693
Stranahan, D. V., M. D. . . . . 60
Wood, Thomas Fanning, M. D. 247

Ophthalmology . . . . . . . . . . 364
Otology . . . . . . . . . 621
Sympathetic Experiments Bearing Upon . . . . . 653
Osteogenesis and Osteoplasty in Crushing Lesions of the Extremities . . . . . . . . . . . . 261
Otorrhea, Symptoms of Cerebral Implications in . . . . . . . . . 622
Ovarian Tumor, a Large . . . . . 364
Oxalic Acid, Action of and its Derivatives on the Animal Economy 556

PAN-AMERICAN Medical Congress . . 45, 53, 494, 503, 641
New By-laws of . . . . . 578
Bulletin . . . . . . . . . . 579

PAGE.
Parmenter, John, M. D., Bruises of the Brain, Surgery of . . 104
Unjust Condemnation of Catgut . . . . . . . . 556
Park, Roswell, M. D., Actinomycosis, with Report of a Case . . . . 325
Papoid in Diphtheria . . . . . . . 11
Pathology and Treatment of Club-foot . . . . . . . . . . . . . . 155
Pelvic Inflammation, Natural History of . . . . . . . . . . . . 33
Pepper, William, M. D., Address by 53

**Personals:**

Abbott, F. W., M. D. . . . . . 631
Banta, Rollin L., M. D. . . . 185
Bartholow, Roberts, M. D. . . 564
Benedict, A. L., M. D. . . . . 695
Bratton, M. D. . . . . . . . 564
Brown, George L. . . . . . . 119
Bull, Louis A., M. D. . . . . 695
Carroll, Evangeline, M. D. . . 694
Cary, Charles, M. D. . . . 564, 694
Clark, Horace, M. D. . . . 310
Coakley, J. B., M. D. . . . . . 694
Crego, F. S., M. D. . . . . 694
Crockett, M. A., M. D. . . . . 694
Davis, W. E. B., M. D. . . . . 120
De Schweinitz, G. E., M. D. . 373
Douglas, Richard, M. D. . . . 374
Duff, John Milton, M. D. . . 439
Dunham, S. A., M. D. . . . . 502
Dwyer, T. F., M. D.. . . . . . 631
Fell, George E., M. D. . . . . 694
Gihon, Albert L., M. D. . . . 56
Gray, Landon Carter, M. D. . 309
Heath, W. H., M. D. . . . . 631
Hoagland, C. N., M. D. . . . 758
Johnston, George Ben., M. D. . 695
Jones, H. Corwin, M. D. . . . 694
Lanphear, Emory, M. D. . . . 692
Lewis, Bransford, M. D. . . . 120
Love, Professor I. N. . . . . . 757
Lydston, G. Frank, M. D. . . 440
McCreary, J. B., Ex-Governor 55
McGuire, Hunter, M. D. . . . 57
McMurtry, Lewis S., M. D. . . 56
Manton, Walter P., M. D. . . 695
Marcy, William L., Esq. . . . 629
Mulford, Henry J., M. D. . . . 57
Mynter, Herman, M. D. . . 631
Palmer, Edward R., M. D. . . 57
Parmenter, John, M. D. . . . 247
Pasteur, Louis . . . . . . . . 502
Pettus, M. D. . . . . . . . . 564
Pleuthner, W. H., M. D. . . . 631
Price, Joseph, M. D. . . . . . 183
Renner, W. S., M. D. . . 373, 564
Rodes, John Edwin, M. D. . . 693
Roe, John O., M. D. . . . . . . 119
Roosa, D. B. St. John, M. D. . 439
Sajous, Charles E., M. D. . . 247
Siegfried, Cyrus, M. D. . . . . 564
Snow, Irving M., M. D. . . . . 695
Starr, Elmer, M. D. . . . . . . 631
M. Allen, M. D. . . . . . . 691
Sternberg, George M., M. D. . 503
Suiter, A. Walter, M. D. . . . 57
Sutton, R. Stansbury, M. D.. . 57
Tremaine, W. S., M. D. . 631, 760
Vander Veer, A., M. D. . . . . 631
Van Sickle, Frederick L., M. D. 694
Verneuil, Prof. . . . . . . . . 502
Watson, Irving A., M. D. . . . 112
Weber, Col., U. S. Commissioner of Immigration . . . 182
Webster, David, M. D. . . . . 760
Wende, Ernest, M. D. 371, 630, 994

Peterson, Frederick, M. D., Epilepsy, Treatment of . . . . 12
Electricity in Nervous Diseases . . . . . . . . . . 147
Physiological Strabismus . . . . . 615
Physician Dispensing His Own Medicine, Advantages of . . . . 681
Pilocarpin, Effects of upon Epileptics . . . . . . . . . . . . . . . 111
Pohlman, Julius, M. D., Physiological Strabismus . . . . . . . 615
Poisoning by Antiseptics . . . . . 559
Potter, William Warren, M. D., Specialism in Medicine as Related to Surgery and Gynecology . . . . 302
Annual Address before the Medical Society of the County of Erie . . . . 401
Pregnancy, Nervous Disturbances of, due to Defective Excretion . 603
Price, Joseph, M. D., Pelvic Inflammation, Natural History of . 33
Prize of the Belgium Academy of Medicine . . . . . . . . . 735
Prizes, The Hodgkins Fund . . . 758
Progressive Muscular Atrophy . . 616

**Progress in Medical Science:**

Bacteriology and Dermatology, Department of . . . . . . . 167
Chemistry . . . . . . . . . 554
Hypodermic Sepsis, Danger of . 51
Internal Medicine . . . . . . 50
Larynx, Ulceration of, Following Intubation . . . . . . . 51
Medicine . . . . . . . . . . . 483
Nervous Diseases . . . . . . 616
Ophthalmology . . . . . . . . 364

PAGE.
Surgery . . . . . . . . . . 104, 556
Typhoid Fever, Chloroform in . 50

Pryor, John H., M. D., Lithemia, Clinical Aspects of . . . . . . . 220
Pterygium . . . . . . . . . . . . 366
Ptosis, A New Method of Operating in . . . . . . . . . . . . 364
Case of Acute Bilateral . . 547
Ptomaines of Infectious Disease . . 555
Puerperal Infection . . . . . . . 17
Sepsis, Prevention of . . . 52
Putnam, James O., Remarks at the Dedication of Buffalo University . . . . . . . 517
James W., M. D., Progressive Muscular Atrophy . 616

QUARANTINE LAW, New National . . . . . . 560
National . . . . . . . . . . 192
Quinine, Double Salts of, etc. . . . 362

RECTAL INJECTIONS . . . . 238
Reed, Charles A. L., M. D., Intra-pelvic Diseases of Women, Early Diagnosis and Early Operation in . . 76
Responsibility of Physician to Patient, Considered from a Legal Point of View . . . . . . . . . 606
Resorcin . . . . . . . . . . . . . 49

**Reviews:**

Adler: Fissure of the Anus and Fistula-in-Ano . . . . . 768
Aulde: Pocket Pharmacy, with Therapeutic Index . . . . 192
Baker: Kirk's Hand-Book of Physiology . . . . . . . . 510
Barr: Cerebro-Spinal Meningitis . . . . . . . . . . . . 313
Baruch: Uses of Water in Modern Medicine . . . . . 313
Bauduy: Diseases of the Nervous System . . . . . . . . . 315
Beach: Histology, Pathology, and Bacteriology . . . . . . 769
Beard: Sexual Neurasthenia . 764
Nervous Exhaustion . . . 764
Beasley: Book of Prescriptions . . . . . . . . . . . . 194
Bocquillon-Limousin: Formulaire des Medicaments Nouveau et des Medications Nouvells pour 1893 . . . . . 576
Boenning: Treatise on Practical Anatomy . . . . . . . . 192

PAGE.
Bosworth: Treatise on Diseases of the Nose and Throat . 252
Boyce: Morbid Histology . . 381
Burnett; American Text-Book of Surgery . . . . . . . 250
System of Diseases of the Eye, Nose and Throat . 765
Canfield: Hygiene of the Sickroom . . . . . . . . . . . . 384
Cathell: Book on the Physician Himself . . . . . . . . 254
Davenport: Diseases of Women . . . . . . . . . . . . . 191
Davis: Principles and Practice of Medicine . . . . . . . . 318
Davidson: Geographical Pathology . . . . . . . . . . . 450
Delafield: Hand-Book of Pathological Anatomy and Histology . . . . . . . . . . . . 507
Dexter: Anatomy of the Peritoneum . . . . . . . . . . . 574
Dulles: Accidents and Emergencies . . . . . . . . . . . 508
Eisenberg: Bacteriological Diagnosis . . . . . . . . . . 68
Ewald: Diseases of the Stomach . . . . . . . . . . . . 313
Flint: Encyclopedia of Medicine and Surgery . . . . . . 67
Foster: Illustrated Encyclopedic Medical Dictionary . . . 190
Fuchs: Ophthalmology . . . 445
Gould: Pocket Medical Dictionary . . . . . . . . . . . 451
Gouley: Diseases of the Urinary Apparatus . . . . . . . 311
Gowers: Manual of Diseases of the Nervous System . . . . . . . . . . . . . 125
Syphilis and the Nervous System . . . . . . . . 768
Gray: Nervous and Mental Diseases . . . . . . . . . 504
Halliburton: Text-Book of Chemical Physiology and Pathology . . . . . . . . . . . 187
Hamilton: Manual of Medical Jurisprudence . . . . . . . 571
Hansell: Manual of Clinical Ophthalmology . . . . . . 575
Hardaway: Manual of Skin Diseases . . . . . . . . . . 575
Hare: System of Practical Therapeutics . . . . . . 63
Text-Book of Practical Therapeutics . . . . . . 705
Herman: First Lines in Midwifery . . . . . . . . . . . 123

PAGE.
Herter: Diagnosis and Disease of the Nervous System . 321
Hummel: Medical Journal Advertising . . . . . . . . . . . 317
Index-Catalogue of the Library of the Surgeon-General's Office, U. S. Army . . . . . 386
Irwin: Hydrotherapy of Saratoga . . . . . . . . . . . . . 447
Jackson: Ready Reference Hand-Book of Diseases of the Skin . . . . . . . . . . . 573
Keating: International Clinics . . . . . . . 66, 379, 448, 767
New Pronouncing Dictionary of Medicine . . . . 193
Kenner: Contributions of Physicians to the English and American Literature . . . . 313
Kirchoff: Hand-Book of Insanity for Practitioners and Students . . . . . . . . . . . . 766
Le Favre: Delsartian Physical Culture . . . . . . . . . . . 259
Leonard's Physician's Pocket Day-Book . . . . . . . . 322
Over 1,000 Prescriptions and Formulæ . . . . . 322
Lusk: Science and Art of Midwifery . . . . . . . . . . . 122
Lydston: Gonorrhea and Urethritis . . . . . . . . . 313
Addresses and Essays . . 510
Lyman: Principles and Practice of Medicine . . . . . . 255
McClellan: Regional Anatomy 120
Mathews: Diseases of the Rectum, Anus, and Sigmoid Flexure . . . . . . . . . . . 442
Moore: Eruptive and Continued Fevers . . . . . . . . . 379
Morris: Human Anatomy . . 761
Morrow: System of Genito-Urinary Diseases, Syphilology and Dermatology . . . . 704
Ormerod: Diseases of the Nervous System . . . . . . . . . 320
Peddis: Manual of Physics . . 385
Physician's Visiting List . . . 383
Potter: Hand-Book of Materia Medica, Pharmacy, and Therapeutics . . . . . . . . . 706
Pozzi: System of Gynecology . 258
Prudden: Drinking Water and Ice Supplies and Their Relations to Health and Disease . 124
Public Ledger Almanac, 1893 . 576
Remondino: Mediterranean Shores of America . . . . . 189

PAGE.
Rickets: What to do in Case of Accident . . . . . . . . . . . 508
Senn: Tuberculosis of Bones and Joints . . . . . . . . 377
Skene: Treatise on the Diseases of Women . . . . . . . . 65
Smith: Naphey's Modern Therapeutics . . . . . . . . . . . . 319
Solis-Cohen: Essentials of Medical Diagnosis . . . . . 258
Stearns: Lectures on Mental Diseases . . . . . . . . . . . . 763
Sunter: All Around the Year 1893 . . . . . . . . . . . 318
Sutton: Surgical Diseases of the Ovaries and Fallopian Tubes . . . . . . . . . . . . 375
Taylor: Manual of Medical Jurisprudence . . . . . . . . 707
Thomson: Outlines of Zoology . . . . . . . . . . . . . 257
Transactions of the American Surgical Association . 706
Southern Surgical and Gynecological Association . . . . . . . . . . . 61
Medical Society of the State of New York, 1892 . 188
Thirteenth Annual Meeting of the American Laryngological Association . . . . . . . . . . . 320
Eighth Annual Meeting of the American Climatological Association . . . 315
Of the Association of American Physicians . . 319
Treat: International Medical Annual and Practitioner's Index . . . . . . . . . . . . 769
Treves: Student's Hand-Book of Surgical Operations . . . 385
Trimble: Practical and Analytical Chemistry . . . . . 323
Tyson: Manual of Physical Diagnosis . . . . . . . . . . 194
Usher: Alcoholism and its Treatment . . . . . . . . . . 572
Vaughan: Ptomaines, Leucomaines, and Bacterial Proteids . . . . . . . . . . . . 321
Walker: Physician's Complete Book of Records . . . . . . 382
Weeks-Shaw: Text-Book of Nursing . . . . . . . . . . 194
Wilson: Hygiene and Sanitary Science . . . . . . . . . 509
International Pocket Visiting List . . . . . . . 378

PAGE.

Richmond, Charles H., M. D., Tumor, Ovarian, of Unusual Size, Removal . . . . . 95
Nelson, G., M. D., Appendicitis, Treatment of, with Illustrative Cases . 1
Riley, Henry A., A. B., LL. B. Medical Jurisprudence, Recent . 299
Roads, Good . . . . . . . . . . . 54
Ross, James F. W., M. D., Ectopic Gestation, Actual Experience Cases . . . . . . . . . . . . . 21

SALANINE, Employment of in Affections of the Stomach . . 111
Semmelweis, J. Ph. Prof. Erection of Monument to His Memory . . 630
Sepsis, Danger of Hypodermic . . 51
Sixth Health District of Buffalo, Report of Condition of . . . . . 173
Smith, Eugene A., M. D., Puerperal Infection . . . . . . 17
Report of a Case of Acute Bilateral Ptosis . . . . 547
Snow, Irving M., M. D., Indigestion and Malnutrition in Nursing Infants . . . . . . . . . . . . 726

**Society Meetings:**

American Medical Association . . . . . . . 636, 698, 701
Social Science Association 58
Electro-Therapeutic Association . . . . . . 185, 639
Microscopical Society . . . . . . . . . . . 58, 640, 701
Public Health Association 59
Association of Obstetricians and Gynecologists . . . . . 118, 186, 565, 740
Orthopedic Association . . . . . . . . . . . 119, 310
Dermatological Association . . . . . . . . . . . 119
Gynecological Society . . 119
Neurological Association . 565
Surgical Association . . . . . . . . . . 566, 690, 697
Association for the Advancement of Science . . . . . . . . 640, 701
Academy of Medicine . . 700
Medical Editors' Association . . . . . . . . 702
Association of Erie Railway Surgeons . . . . . . . . . 48
Association, Mississippi Valley Medical . . . . . . . . . . 760

PAGE.

Buffalo Microscopical Club . . . . . 250, 310, 503, 565, 701
Academy of Medicine . . . . . . . . . 310, 503, 504
Conference of State Medical Examining and Licensing Boards . . . . . . . . . . 638
Erie County Medical Society . 701
French Society of Electropathy . . . . . . . . . . . . 565
International Dermatological Congress . . . . . . . . 118
Congress, Eleventh . 249, 639
Lake Erie Medical Society . . 59
Medical Society of the County of Erie . . . . . . 374, 401
Society of the State of New York . . . . . 374, 440, 504
Association of Georgia . . 565
Association of Central New York . . . . . . . 701
National Association of Railway Surgeons . . . . . . . . 374
New York State Association of Railway Surgeons . . . . . 186
Pan-American Medical Congress . . . . . . . . . 370
Section of General Surgery . . . . . . . . . 761
Southern Surgical and Gynecological Association . . 248, 307

**Society Proceedings:**

American Association of Obstetricians and Gynecologists . . . . . . . . . . . . . 235, 740
Buffalo Academy of Medicine . . . . . . . . . 215, 235, 350, 610
Medical Society of the County of Erie . . . . . . . . . 39, 424
Philadelphia County Medical Society . . . . . . . . . . 352

Spore and Non-spore Forming Pathogenic Bacteria . . . . . . . 168
Stanbro, Frederick H., M. D., The Careless Examination of Urine . 736
Sterilization of Water by Heat . . 239
Steel, Extraction of from Interior of Eye . . . . . . . . . . . . . . . 387
Stockton, Charles G., M. D., Lithemia, Digestive Disturbances in . 229
Storrs, Caryl B., M. D., Advantages of Physician Dispensing His Own Medicine . . . . . . . . . . . 681
M., M. D., Maxillary Nerve, Removal of . . 293

PAGE.
Strabismus, Physiological . . . . . 615
Sulfonal Poisoning, Symptoms of . 489
Suppuration, Complicating Tuberculous Disease of the Bones and Joints, Management of . . . 598, 680
Syphilis and Marriage . . . . . . 417
Hypodermic Treatment of 167
and Rickets, Clinical Remarks, Congenital . . . 411
Syrup of Hydriodic Acid and its Uses . . . . . . . . . . . . . . 486

TENDON REFLEXES, Behavior of in Health and Disease . 133
Tenement Houses . . . . . . . . 177
Tetanus Bacillus . . . . . . . . . 167
Thornbury, F. J., M. D., Internal Medicine.—Wound Treatment, Present Status of.—Bacteriology and Dermatology . . . 50, 92, 167
Topics of the Month . 53, 112, 181, 244, 307, 370, 435, 499, 562, 629, 691, 757
Tracoma, Treatment of by Expression . . . . . . . . . . . . . . . 622
Trional as a Hypnotic, Value of . . 483
Tumor of the Cerebrum, Two Cases of . . . . . . . . . . 619
Ovarian, of Unusual Size, Removal of . . . . . . . 95
Typhoid Fever, Chloroform in . . 50

UNDERGARMENTS, to Wash Silk . . . . . . . . . . . . 131
United States Investor, Prizes Offered by . . . . . . . 244

PAGE.
University of Buffalo, Dedication and Opening of New Building . . . . . . . . 517
History of Present Standing of . . . . . . . . . 519
Tennessee, College Commencement . . . . . . 562
Urine, Careless Examination of . . 736
Uterine Appendages. Results of Removal of . . . . 105
Cancer, Nature and Choice of Treatment of . . . . 653

VACCINATION . . . . . . . 557
Vermiform Appendix, Present Status of . . . . . . . . . . 106
Vertebral Fractures . . . . . . . 625
Vomiting, Hydrochloric Acid in . . 479

WEIGEL, L A., M. D., Suppuration, Complicating Tuberculous Disease of the Bones and Joints, Management of . . . . . . . 596
Wende, Ernest, M. D., Cholera, Precautions Against . . . . . . 176
Williams, Herbert U., M. D., A Shield to Protect the Clinical Thermometer . . . . . . . . . . 683
Woman's Russet Shoes . . . . . 132
Woodbury, Frank, M. D., Carica Papaya in Gastro-intestinal Disorders . . . . . . . . . . . . 97
World's Columbian Exposition, Section of the Congress . . . . . 512
Wound Treatment, Present Status of . . . . . . . . . . 92
Of the Heart . . . . . . 557

